# Item-Score Reliability as a Selection Tool in Test Construction

**DOI:** 10.3389/fpsyg.2018.02298

**Published:** 2019-01-11

**Authors:** Eva A. O. Zijlmans, Jesper Tijmstra, L. Andries van der Ark, Klaas Sijtsma

**Affiliations:** ^1^Methodology and Statistics, Tilburg University, Tilburg, Netherlands; ^2^Research Institute of Child Development and Education, University of Amsterdam, Amsterdam, Netherlands

**Keywords:** correction for attenuation, corrected item-total correlation, item-score reliability, item selection in test construction, method CA, method λ_6_, method MS

## Abstract

This study investigates the usefulness of item-score reliability as a criterion for item selection in test construction. Methods MS, λ_6_, and CA were investigated as item-assessment methods in item selection and compared to the corrected item-total correlation, which was used as a benchmark. An ideal ordering to add items to the test (bottom-up procedure) or omit items from the test (top-down procedure) was defined based on the population test-score reliability. The orderings the four item-assessment methods produced in samples were compared to the ideal ordering, and the degree of resemblance was expressed by means of Kendall's τ. To investigate the concordance of the orderings across 1,000 replicated samples, Kendall's *W* was computed for each item-assessment method. The results showed that for both the bottom-up and the top-down procedures, item-assessment method CA and the corrected item-total correlation most closely resembled the ideal ordering. Generally, all item assessment methods resembled the ideal ordering better, and concordance of the orderings was greater, for larger sample sizes, and greater variance of the item discrimination parameters.

## 1. Introduction

Measurements obtained by tests are only trustworthy if the quality of the test meets certain standards. Reliability is an important aspect of test quality that is routinely reported by researchers (e.g., AERA et al., 2014) and expresses the repeatability of the test score (e.g., Sijtsma and Van der Ark, in press). It is important that tests, for example when used in the psychological domain, are reliable. When adapting an existing test, the test constructor may wish to increase or decrease the number of items for various reasons. On the one hand, the existing test may be too short, resulting in test-score reliability that is too low. In this case, adding items to the test may increase test-score reliability. On the other hand, the existing test may be too long to complete in due time. A solution could be to decrease the number of items, but after removal of a number of items, the test score based on the remaining items must be sufficiently reliable. Test constructors could use the reliability of individual items to make decisions about the items to add to the test or to remove from the test. This article investigates the usefulness of item-score reliability methods for making informed decisions about items to add or remove when adapting a test.

Several approaches to item selection in test construction have been investigated. Raubenheimer (2004) investigated an item selection procedure that maximizes coefficient alpha of each subscale within a multi-dimensional test, and simultaneously maximizes both the convergent and discriminant validity using exploratory factor analysis. Raykov (2007) and Raykov (2008) discussed the use of procedure “alpha if item deleted” to omit items from a test and concluded that maximizing coefficient alpha results in loss of criterion validity. Erhart et al. (2010) studied item reduction by either maximizing coefficient alpha or the item fit of the partial credit model (Masters, 1982). They concluded that both item reduction approaches should be accompanied by additional analyses. Because the quality of a test depends on more than only the reliability of its test score, taking additional information in consideration obviously is a wise strategy. However, in this study we preferred to focus on optimizing test-score reliability in the process of adding items to the test or omitting items from the test. This enabled us to assess the value of particular item selection procedures and item-assessment methods, in particular, item-score reliability methods. The usability of item-score reliability in item selection procedures was investigated in detail.

Zijlmans et al. (2018b) investigated four methods to estimate item-score reliability. The three most promising methods from this study were methods MS, λ_6_, and CA. Zijlmans et al. (2018a) applied these methods to empirical data sets and investigated which values of item-score reliability can be expected in practice and how these values relate to four other item indices that did not assess the item-score reliability in particular, which are item discrimination, item loadings, item scalability, and corrected item-total correlations. In a third study (Zijlmans et al., submitted), the relationship between the item-score reliability methods and the four other item indices was further investigated by means of a simulation study. The use of the three item-score reliability methods for maximizing test-score reliability has not been investigated yet. Therefore, in this study the usefulness of item-score reliability methods MS, λ_6_, and CA for constructing reliable tests was investigated.

The three research questions we addressed are the following. First, are item-score reliability methods useful for adding items to a test or omitting from a test, when the goal is to maximize test-score reliability of the resulting test? Second, to what extent do the orderings in which the three item-score reliability methods select items resemble the theoretically optimal ordering in which items are selected or removed when maximizing population test-score reliability? Third, do the orderings produced by each of the three item-score reliability methods bear more resemblance to the theoretically optimal ordering than the ordering the corrected item-total correlation produced? These questions were addressed by means of a simulation study.

This article is organized as follows. First, we discuss bottom-up and top-down procedures for constructing a test. Second, we discuss the item-assessment methods we used, which are item-score reliability methods MS, λ_6_, and CA, and the corrected item-total correlation. Third, we discuss the design for the simulation study and the data-generating process. Finally, the results and their implications for test construction are discussed.

## 2. Item Selection in Test Construction

In this study, we focus on two procedures for test construction. For both procedures, the test constructor has to make an informed decision about the balance between the desired length of the test and the desired minimum test-score reliability. Hence, we focus entirely on selection or omission of items based on formal assessment methods. The first procedure selects items from the pool of available items, and adds the selected items one by one to the preliminary test. We refer to this procedure as the bottom-up procedure. The second procedure uses the complete pool of available items as the initial test, and selects items one by one for elimination from this test. We refer to this procedure as the top-down procedure.

### 2.1. Bottom-Up Procedure

The bottom-up procedure starts by defining an initial test consisting of two items from the pool of available items. In general, and apart from the present study, different criteria to select the initial two-item test can be used. For example, the test constructor may consider the two items she starts with the substantive kernel of the test, or she may choose the two items that have proven to be of excellent quality in the past. In both examples, the researcher includes the item in the test. In our study, the item pair having the highest test-score reliability was selected. The selected item pair constituted the initial test and both items were removed from the pool of available items. In the next step, the third item was added to the two-item test that maximizes the test-score reliability $\rho_{XX^{\prime }}$ for a three-item test, based on all available choices; then, the fourth item was added to the three-item test following the same logic, and so on. In practice, it is impossible to estimate $\rho_{XX^{\prime }}$, because parallel test scores *X* and *X*′ are usually unavailable (Lord and Novick, 1968, p. 106). In this study, four item-assessment methods were investigated that can be used to add the items to the test. The test constructor may use one of these four item-assessment methods to continue the bottom-up procedure until the test has the desired length or a sufficiently high test-score reliability, or both. Because in practice, different test constructors may entertain different requirements for test length and minimum reliability, adding items to the preliminary test may stop at different stages of the procedure. Hence, for the sake of completeness, we described the complete ordering based on adding each of the available items to the test until all items were selected.

### 2.2. Computational Example Bottom-Up Procedure

We discuss a computational example. We started with 20 equally difficult items for which the item discrimination parameters were ordered from smallest to largest. To select the initial 2-item test, we considered the theoretical $\rho_{XX^{\prime }}$-values for all possible 2-item tests. Test-score reliability was defined theoretically based on available item parameters of the two-parameter logistic model (2PLM), assuming a standard normal distribution of the latent variable (see simulation study). Items 19 and 20 had the highest test-score reliability, so this pair constituted the initial test; see Table 1. In Step 1, a pool of 18 items was available from which to add an item to the preliminary test version. Consider the columns in Table 1 headed by Step 1. The $\rho_{XX^{\prime }}$ column shows the $\rho_{XX^{\prime }}$-values for each 3-item test including one of the available items, so that one can evaluate the test-score reliability of each 3-item test. Item 18 resulted in the highest test-score reliability and was added in Step 2. The procedure continued until the pool of available items was empty. In the penultimate step, which is Step 18, item 2 was added to the preliminary test. Item 1 was added in the last step. From this procedure, we derived the ordering in which the items were added to the preliminary test versions, when the goal was to maximize the test-score reliability in each step.

**Table 1:** Example item-selection procedure following the bottom-up procedure based on the test-score reliability $\rho_{XX^{\prime }}$.

**Step 1**			**Step 2**			**⋯ **	**Step 17**			**Step 18**		
**Items in**	**Items in**	$\rho_{XX^{\prime }}$ **if**	**Items in**	**Items in**	$\rho_{XX^{\prime }}$ **if**		**Items in**	**Items in**	$\rho_{XX^{\prime }}$ **if**	**Items in**	**Items in**	$\rho_{XX^{\prime }}$ **if**
**test**	**pool**	**item added**	**test**	**pool**	**item added**	⋯	**test**	**pool**	**item added**	**test**	**pool**	**item added**
20	1	0.462	20	1	0.556	⋯	20	1	0.807	20	1	0.809
19	2	0.467	19	2	0.560	⋯	19	2	0.808	19	**2**	**0.810**
	3	0.473	18	3	0.564	⋯	18	**3**	**0.808**	18		
	4	0.479		4	0.568	⋯	17			17		
	5	0.484		5	0.573	⋯	16			16		
	6	0.491		6	0.577	⋯	15			15		
	7	0.497		7	0.582	⋯	14			14		
	8	0.503		8	0.586	⋯	13			13		
	9	0.510		9	0.591	⋯	12			12		
	10	0.516		10	0.595	⋯	11			11		
	11	0.523		11	0.600	⋯	10			10		
	12	0.530		12	0.605	⋯	9			9		
	13	0.536		13	0.610	⋯	8			8		
	14	0.543		14	0.615	⋯	7			7		
	15	0.550		15	0.620	⋯	6			6		
	16	0.557		16	0.625	⋯	5			5		
	17	0.564		**17**	**0.630**	⋯	4			4		
	**18**	**0.571**				⋯				3		

*The example is based on the condition with small variance of discrimination parameters. The $\rho_{XX^{\prime }}$ column indicates the possible test-score reliability, if in the next step, that item would be added to the test. For each step the selected item is indicated in boldface*.

### 2.3. Top-Down Procedure

For the top-down procedure, the complete pool of available items constitutes the initial test, and the items are deleted one by one until two items remain. Ideally, the test-score reliability of all twenty 19-item tests is computed, determining which item should be omitted, so that the test consisting of the remaining 19 items had the highest test-score reliability of all 19-item tests. The first item that is omitted either increases the test-score reliability the most or decreases the test-score reliability the least. This procedure is repeated until the test consisted of only two items. Two items constitute the minimum, because an item-assessment method cannot be applied to a single item. This procedure results in an ordering in which items were omitted from the test. A test constructor usually does not continue until the test consists of only two items but rather stops when the resulting test has the desired length or the desired test-score reliability, or both. In our study, for the sake of completeness, we continued the item selection until the test consisted of only two items so that the results for the complete top-down procedure were visible.

### 2.4. Computational Example Top-Down Procedure

We employed the same 20 items we used in the computational example for the bottom-up procedure, and included all items in the initial test. In Step 1, $\rho_{XX^{\prime }}$ was computed when a particular item was omitted from the test (using the procedure outlined in the simulation study section; see Table 2, column $\rho_{XX^{\prime }}$ if item omitted for the $\rho_{XX^{\prime }}$-values). In the example, omitting item 1 from the test produced the highest $\rho_{XX^{\prime }}$-value. Thus, item 1 was omitted and we continued with the 19-item test. In Step 2, omitting item 2 resulted in the highest test-score reliability for the remaining 18 items. This procedure was repeated until the test consisted of only items 19 and 20.

**Table 2:** Example item-selection procedure following the top-down procedure based on the test-score reliability $\rho_{XX^{\prime }}$.

**Step 1**			**Step 2**			**⋯ **	**Step 17**			**Step 18**		
**Items**	**Items in**	$\rho_{XX^{\prime }}$ **if**	**Items**	**Items in**	$\rho_{XX^{\prime }}$		**Items**	**Items in**	$\rho_{XX^{\prime }}$	**Items**	**Items in**	$\rho_{XX^{\prime }}$
**omitted**	**test**	**item omitted**	**omitted**	**test**	**item omitted**	⋯	**omitted**	**test**	**item omitted**	**omitted**	**test**	**item omitted**
	**1**	**0.810**	1	**2**	**0.808**	⋯	1	**17**	0.571	1	**18**	**0.481**
	2	0.809		3	0.807	⋯	2	18	0.564	2	19	0.47
	3	0.809		4	0.807	⋯	3	19	0.557	3	20	0.46
	4	0.808		5	0.806	⋯	4	20	0.550	4		
	5	0.808		6	0.806	⋯	5			5		
	6	0.807		7	0.805	⋯	6			6		
	7	0.806		8	0.804	⋯	7			7		
	8	0.806		9	0.804	⋯	8			8		
	9	0.805		10	0.803	⋯	9			9		
	10	0.804		11	0.802	⋯	10			10		
	11	0.804		12	0.801	⋯	11			11		
	12	0.803		13	0.800	⋯	12			12		
	13	0.802		14	0.799	⋯	13			13		
	14	0.801		15	0.799	⋯	14			14		
	15	0.800		16	0.798	⋯	15			15		
	16	0.800		17	0.797	⋯	16			16		
	17	0.799		18	0.796	⋯				17		
	18	0.798		19	0.795	⋯						
	19	0.797		20	0.794	⋯						
	20	0.796				⋯						

*The example is based on the condition with small variance of discrimination parameters. The $\rho_{XX^{\prime }}$ column indicates the possible test-score reliability, if that item would be omitted from the test in the next step. For each step the selected item is indicated in boldface*.

### 2.5. Item-Assessment Methods

We used the three item-score reliability methods MS, λ_6_, and CA, and the corrected item-total correlation to add items to the preliminary test or to omit items from the preliminary test. The corrected item-total correlation was included to compare the item-score reliability methods to a method that has been used for a long time in test construction research. Both the bottom-up and the top-down procedures were applied using the four item-assessment methods instead of test-score reliability $\rho_{XX^{\prime }}$. The eight orderings that resulted from combining the two item selection procedures with the four item-assessment methods were compared to the ordering based on the theoretical test-score reliability, to infer which item-assessment method resembled the ordering that maximizes the test-score reliability best.

### 2.6. Item-Score Reliability Methods

The following definitions were used (Lord and Novick, 1968, p. 61). Test score *X* is defined as the sum of the *J* item scores. Let *X*_*i*_ be the item score, indexed *i* (*i* = 1, …, *J*); $X=\sum_{i=1}^{J}X_{i}$. Item-score reliability is defined as the ratio of the true-score variance, denoted $\sigma_{T_{i}}^{2}$, and the observed-score variance, denoted $\sigma_{X_{i}}^{2}$. The observed-score variance can be split in true-score variance and error variance denoted $\sigma_{E_{i}}^{2}$, which means item-score reliability can also be defined as 1 minus the proportion of observed-score variance that is error variance; that is,

(1) $$\rho_{ii^{\prime }}=\frac{\sigma_{T_{i}}^{2}}{\sigma_{X_{i}}^{2}}=1-\frac{\sigma_{E_{i}}^{2}}{\sigma_{X_{i}}^{2}}.$$

Three methods to approximate item-score reliability were used to decide which item will be added to the test or omitted from the test: method MS, method λ_6_, and method CA. These methods are briefly discussed here; see Zijlmans et al. (2018b) for details.

#### 2.6.1. Method MS

Method MS is based on the Molenaar-Sijtsma test-score reliability method (Sijtsma and Molenaar, 1987; Molenaar and Sijtsma, 1988). This method uses the double monotonicity model for dichotomous items proposed by Mokken (1971) which assumes a unidimensional latent variable θ, locally independent item scores, and monotone nondecreasing, and nonintersecting item-response functions. The items are ordered from most difficult to easiest and this ordering is used to obtain an approximation of an independent replication of the item of interest, denoted *i*. Mokken (1971, pp. 142-147) proposed to approximate independent replications of the item scores by using information from the item of interest, the next-easier item *i*+1, the next more-difficult item *i*−1, or both neighbor items. The idea is that items that are close to the item of interest in terms of location provide a good approximation of an independent replication of the target item. We denote method MS for estimating item-score reliability $\rho_{ii^{\prime }}^{MS}$ and estimate the independent replication approximated in $\rho_{ii^{\prime }}^{MS}$ using the procedure as explained by Van der Ark (2010).

#### 2.6.2. Method λ_6_

Test-score reliability method λ_6_ (Guttman, 1945) was adjusted by Zijlmans et al. (2018b), such that it approximates the reliability of an item score. Let $\epsilon_{i}^{2}$ be the residual error variance from the multiple regression of the score on item *i* on the remaining *J*−1 item scores. The ratio of $\epsilon_{i}^{2}$ and the observed item variance $\sigma_{i}^{2}$ is subtracted from unity to obtain the item-score reliability estimate by means of method λ_6_, denoted $\rho_{ii^{\prime }}^{\lambda_{6}}$; that is,

(2) $$\rho_{ii^{\prime }}^{\lambda_{6}}=1-\frac{\epsilon_{i}^{2}}{\sigma_{X_{i}}^{2}}.$$

#### 2.6.3. Method CA

Method CA is based on the correction for attenuation (Lord and Novick, 1968, pp. 69-70; Nunnally and Bernstein, 1994, p. 257; Spearman, 1904) and correlates the item score with a test score, which is assumed to measure the same attribute as the item score (Wanous and Reichers, 1996). This test score can be obtained from the remaining *J*−1 items in the test or the items in a different test, which is assumed to measure the same attribute as the target item. We denote item-score reliability approximated by method CA as $\rho_{ii^{\prime }}^{CA}$. The corrected item-total correlation is defined as ρ_*X*_*i*_*R*_(*i*)__, and correlates the item score with the rest score, defined as *R*_(*i*)_ = *X*−*X*_*i*_. Coefficient α_*R*_(*i*)__ is a lower bound to the reliability of the rest score, estimated by reliability lower bound coefficient α (e.g., Cronbach, 1951). Method CA is defined as

(3) $$\rho_{ii^{\prime }}^{CA}=\frac{\rho_{X_{i}R_{\left(i\right)}}^{2}}{\alpha_{R_{\left(i\right)}}}.$$

Earlier research (Zijlmans et al., 2018b) showed that methods MS and CA had little bias. Method λ_6_ produced precise results, but underestimated ρ*ii*′, suggesting it is a conservative method. These results showed that the three methods are promising for estimating ρ*ii*′.

### 2.7. Corrected Item-Total Correlation

The corrected item-total correlation ρ_*X*_*i*_*R*_(*i*)__ was defined earlier. Higher corrected item-total correlations in a test result in a higher value of coefficient α (Lord and Novick, 1968, p. 331). In test construction, the corrected item-total correlation is used to define the association of the item with the total score on the other items. The corrected item-total correlation is also used by method CA (see Equation 3).

## 3. Simulation Study

By means of a simulation study it was investigated whether the four item-assessment methods added items to a test (bottom-up procedure) or omitted items from a test (top-down procedure) in the same ordering that would result from adding items or omitting items, such that the theoretical $\rho_{XX^{\prime }}$ was maximized in each item selection step.

### 3.1. Method

For the bottom-up procedure, for each item-assessment method, we investigated the ordering in which items were added. In each selection step, the item was selected that had the greatest estimated item-score reliability or the greatest corrected item-total correlation based on its inclusion in the preliminary test. For the top-down procedure, for each item-assessment method, we investigated the ordering in which items were omitted, this time in each step omitting the item that had the smallest item-score reliability or the smallest corrected item-total correlation. The ordering in which items were added or omitted was compared to the ideal ordering if theoretical test-score reliability was used. The degree to which the orderings produced by an item-assessment method resembled the ideal ordering, was expressed by Kendall's τ. The concordance of orderings produced by each item-assessment method over samples was expressed by means of Kendall's *W*. Next, we discuss the details of the simulation study.

Dichotomous scores for 20 items were generated using the 2PLM (Birnbaum, 1968). Let θ be the latent variable representing a person's attribute, α_*i*_ the discrimination parameter of item *i*, and β_*i*_ the location parameter of item *i*. The 2PLM is defined as

(4) $$P(X_{i}=1|\theta )=\frac{\text{exp}\left[\alpha_{i}(\theta -\beta_{i})\right]}{1+\text{exp}\left[\alpha_{i}(\theta -\beta_{i})\right]}.$$

The variance of the discrimination parameters was varied. The discrimination parameter of an item conceptually resembles its item-score reliability (Tucker, 1946). All sets of discrimination parameters had the same median value. We used sets of values that had the same mean on the log scale, which guaranteed that all discrimination parameters were positive and that for each condition the median discrimination parameter was 1, and considered values equidistantly spaced ranging from −0.5 to 0.5, −1 to 1, or −2 to 2 on the log scale. The variance of the discrimination parameters is referred to as either *small*, ranging from 0.61 to 1.65 on the original scale, *average*, ranging from 0.37 to 2.72, or *large*, ranging from 0.14 to 7.39. For all items, the location parameter β_*i*_ had a value of 0. We did not vary β_*i*_, because this would complicate the simulation design, rendering the effect of item discrimination, approximating item-score reliability, on the item selection process harder to interpret. Table 3 shows the item parameters that were used to generate the item scores. Next to the bottom-up and top-down procedures, we varied the sample size *N*. We generated item scores for either a small sample (*N* = 200) or a large sample (*N* = 1, 000). These choices resulted in 2 (sample sizes) × 3 (variances of discrimination parameters) = 6 design cells. In each design cell, 1, 000 data sets were generated. The 1, 000 data sets in each cell were analyzed by the two item selection procedures, each using the four item-assessment methods.

**Table 3:** Item Parameters used to Generate the Item Scores.

**Item no**.	**Small variance of α**	**Average variance of α**	**Large variance of α**	**β**
Item 1	0.61	0.37	0.14	0
Item 2	0.64	0.41	0.17	0
Item 3	0.67	0.45	0.21	0
Item 4	0.71	0.50	0.25	0
Item 5	0.75	0.56	0.31	0
Item 6	0.79	0.62	0.39	0
Item 7	0.83	0.69	0.48	0
Item 8	0.88	0.77	0.59	0
Item 9	0.92	0.85	0.73	0
Item 10	0.97	0.95	0.90	0
Item 11	1.03	1.05	1.11	0
Item 12	1.08	1.17	1.37	0
Item 13	1.14	1.30	1.69	0
Item 14	1.20	1.45	2.09	0
Item 15	1.27	1.61	2.58	0
Item 16	1.34	1.78	3.18	0
Item 17	1.41	1.98	3.93	0
Item 18	1.48	2.20	4.85	0
Item 19	1.56	2.45	5.99	0
Item 20	1.65	2.72	7.39	0

*α = discrimination parameter, β = location parameter. The sets of discrimination parameters had the same mean, and contain values equidistantly. spaced, ranging from -0.5 to 0.5, -1 to 1, and -2 to 2 on the log scale, respectively*.

From the parameters of the data generating model, the ideal ordering for both the bottom-up procedure and the top-down procedure was determined using test-score reliability $\rho_{XX^{\prime }}$ (see simulation study for the procedure). The goal was to maximize $\rho_{XX^{\prime }}$ in each step of the two procedures. For the bottom-up and the top-down procedures, we determined the ideal order to add items to the test or omit items from the test, based on maximizing the theoretical $\rho_{XX^{\prime }}$ in every step. The two item selection procedures and the three sets of discrimination parameters resulted in six ideal orderings. Because discrimination parameters increased going from item 1 to item 20, and the item ordering did not differ over the sets of discrimination parameters, only two ideal item orderings were different. Consequently, for the item selection we have two ideal orderings, one for the bottom-up procedure (consecutively adding items 18, 17, …, 1) and one for the top-down procedure (consecutively omitting items 1, 2, …, 18).

The agreement between the ordering determined by each of the item-assessment methods and the ideal ordering determined by $\rho_{XX^{\prime }}$ was expressed in each data set by means of Kendall's τ. Kendall's τ ranges from −1 to 1, a large negative value indicating that the orderings are dissimilar and a large positive value indicating that the orderings are similar. The item ranks produced by the item-assessment methods can be displayed as a vector, and so can the ideal rank defined at the population level. When the ranks for both elements in a pair agreed this was defined as a concordant pair (*C*), otherwise the pair was discordant (*D*). The total number of pairs equals *n*(*n*−1)/2, where *n* is the length of the vectors. Kendall's τ is defined as

(5) $$\tau =\frac{C-D}{n(n-1)/2}.$$

In our study, *n* = 18, based on 18 item selection steps for both item selection methods. We computed the mean for the 1, 000 Kendall's τ-values obtained in each simulation condition, for every combination of item selection procedure and item-assessment method. The mean quantified the resemblance between the ordering each of the item-assessment methods produced and the ideal ordering.

To investigate how much the orderings the item-assessment methods produced differ over 1, 000 data sets, we computed Kendall's coefficient of concordance, *W*. Kendall's *W* expresses the level of agreement between multiple orderings, and *W* ranges from 0 to 1, a higher value indicating that the orderings an item-assessment method produced are more consistent, resulting in smaller variation. Suppose that item *i* is given rank *r*_*ij*_ in data set *j*, where there are in total *n* ranks and *m* data sets. Then the total rank of object *i* is $R_{i}=\overset{m}{\underset{j=1}{\sum }}r_{ij}$ and the mean value of these ranks is $\bar{R}=\frac{1}{n}\overset{n}{\underset{i=1}{\sum }}R_{i}$. The sum of squared deviations, *S*, is defined as $S=\overset{n}{\underset{i=1}{\sum }}{\left(R_{i}-\bar{R}\right)}^{2}$. Kendall's *W* is defined as

(6) $$W=\frac{12S}{m^{2}(n^{3}-n)}.$$

The number of objects *n* in our study was the number of item selection steps, which was 18. The number of data sets *m* equaled 1, 000. For every simulation condition and every item selection method, Kendall's *W* expressed the agreement among orderings produced by each of the item-assessment methods.

### 3.2. Deriving Item-Score Reliability and Test-Score Reliability From 2PLM

Test-score reliability was defined theoretically based on available item parameters of the 2PLM, assuming a standard normal latent variable, using the following procedure that we briefly outline. Let item score *X*_*i*_ have *m*+1 different values 0, 1, …, *m*. The 2PLM models dichotomous items; hence *m* = 1. Let α_*i*_ denote the discrimination parameter and let β_*i*_ denote the location parameter. In the 2PLM, *P*(*X*_*i*_ = 1|θ)≡*P*_*iθ*_ is modeled as

(7) $$P_{i\theta }=\frac{\text{exp}\left[\alpha_{i}(\theta -\beta_{i})\right]}{1+\text{exp}\left[\alpha_{i}(\theta -\beta_{i})\right]}.$$

The first partial derivative of *P*_*iθ*_ with respect to θ equals

(8) $$P_{i\theta }^{\prime }=\frac{\partial P_{i\theta }}{\partial \theta }=\alpha_{i}P_{i\theta }\left(1-P_{i\theta }\right)$$

(e.g., Baker, 1992, p. 81). Latent variable θ and true score *T* are related. Let *T*_*iθ*_ denote the item true score given a latent trait value. For the classical test theory model, by definition, *T*_*iθ*_ = *E*(*X*_*i*_|θ) (Lord and Novick, 1968, p. 34). Furthermore, using straightforward algebra, it follows that $E\left(X_{i}|\theta \right)=\overset{m}{\underset{x=1}{\sum }}P\left(X_{i}\geq x|\theta \right)$, which reduces to *E*(*X*_*i*_|θ) = *P*_*iθ*_ for *m* = 1. Hence, in the 2PLM we find that *T*_*iθ*_ = *P*_*iθ*_ (Lord, 1980, p. 46). Let $\sigma_{T_{i}}^{2}$ denote the variance of *T*_*i*_. Following the delta method (e.g., Agresti, 2002, pp. 577–581), we can derive that

(9) $$\sigma_{T_{i}}^{2}\approx {\left(P_{i\mu_{\theta }}^{\prime }\right)}^{2}\sigma_{\theta }^{2}.$$

Inserting the right-hand side of Equation 7, in which θ has been replaced by μ_θ_, into Equation 8, and subsequently inserting the right-hand side of Equation 8 into Equation 9 yields

(10) $$\begin{array}{l} \sigma_{T_{i}}^{2}\approx \alpha_{i}^{2}{\left(\frac{\text{exp}[\alpha_{i}(\mu_{\theta }-\beta_{i})]}{1+\text{exp}[\alpha_{i}(\mu_{\theta }-\beta_{i})]}\right)}^{2}\\ \;{\left(1-\frac{\text{exp}[\alpha_{i}(\mu_{\theta }-\beta_{i})]}{1+\text{exp}[\alpha_{i}(\mu_{\theta }-\beta_{i})]}\right)}^{2}\sigma_{\theta }^{2}. \end{array}$$

Let G(θ) be the distribution of latent variable θ, with mean μ_θ_ and variance $\sigma_{\theta }^{2}$. Let $P_{i}\equiv P\left(X_{i}=1\right)=\int_{\theta }\;P_{i\theta }dG\left(\theta \right)$. Let $\sigma_{X_{i}}^{2}$ denote the variance of *X*_*i*_. Because *X*_*i*_ is dichotomous,

(11) $$\sigma_{X_{i}}^{2}=P_{i}(1-P_{i})=\int_{\theta }P_{i\theta }dG(\theta )\left(1-\int_{\theta }P_{i\theta }dG(\theta )\right).$$

Let *P*_*ij*_ ≡*P*(*X*_*i*_ = 1, *X*_*j*_ = 1). Due to the local independence assumption of the 2PLM, one can derive that $P_{ij}=\int_{\theta }\;P_{i\theta }P_{j\theta }dG\left(\theta \right)$. Let σ_*X*_*i*_, *X*_*j*__ denote the covariance between *X*_*i*_ and *X*_*j*_. In classical test theory, σ_*X*_*i*_, *X*_*j*__ = σ_*T*_*i*_, *T*_*j*__ for *i* ≠ *j*. For dichotomous *X*_*i*_ we derive

(12) $$\begin{array}{l} \sigma_{X_{i},X_{j}}=\sigma_{T_{i},T_{j}}=P_{ij}-P_{i}P_{j}=\int_{\theta }P_{i\theta }P_{j\theta }dG(\theta )\\ \;-\int_{\theta }P_{i\theta }dG(\theta )\int_{\theta }P_{j\theta }dG(\theta ). \end{array}$$

Let

(13) $$\sigma_{T}^{2}=\sum_{i=1}^{J}\sigma_{T_{i}}^{2}+\underset{i\neq j}{\sum_{i=1}^{J}\overset{J}{\underset{j=1}{\sum }}}\sigma_{T_{i},T_{j}}$$

and

(14) $$\sigma_{X}^{2}=\sum_{i=1}^{J}\sigma_{X_{i}}^{2}+\underset{i\neq j}{\sum_{i=1}^{J}\overset{J}{\underset{j=1}{\sum }}}\sigma_{X_{i},X_{j}}$$

denote the true-score variance and test-score variance, respectively; the item variances and covariances can be derived from the 2PLM using Equations 10, 11, and 12.

Item-score reliability $\rho_{ii^{\prime }}=\frac{\sigma_{T_{i}}^{2}}{\sigma_{X_{i}}^{2}}$ can be obtained from Equation 10 and Equation 11. Test-score reliability $\rho_{XX^{\prime }}=\frac{\sigma_{T}^{2}}{\sigma_{X}^{2}}$ can be obtained from Equation 13 and Equation 14.

### 3.3. Results

For the bottom-up procedure (upper part of Table 4) and the top-down procedure (lower part of Table 4), for the six design conditions of sample size and variance of discrimination parameters, Table 4 shows the mean Kendall's τ between the ideal ordering and the ordering produced by each of the four item-assessment methods. The standard deviation is shown in parentheses. For every cell, the distribution of Kendall's τ values was inspected visually, and we concluded that the values were approximately normally distributed. In the condition with a small sample size and small variance of the discrimination parameters, mean Kendall's τ ranged from 0.44 for method MS to 0.59 for method CA and the corrected item-total correlation. For both procedures, a larger sample size resulted in a higher mean τ-value. Mean Kendall's τ increased as variance of discrimination parameters increased for both item selection procedures. For a large sample size and large variance of discrimination parameters, mean τ-values ranged from 0.80 for method MS to 0.96 for method CA and the corrected item-total correlation. For both procedures, method CA and the corrected item-total correlation showed the highest mean τ-values, meaning that these item-assessment methods resembled the ordering based on $\rho_{XX^{\prime }}$ best. These two item assessment-methods showed numerically equal mean τ-values, which resulted from the nearly identical orderings method CA and the corrected item-total correlation produced. For the bottom-up procedure, four out of six conditions showed exactly the same ordering for each replication using either method CA or the corrected item-total correlation. For the top-down procedure, this result was found in two out of six conditions. Overall, method MS performed worst of all item-assessment methods, where the difference in τ- values with the other item-assessment methods was smaller for a larger sample size and increasing variance of the discrimination parameters.

**Table 4:** Mean Kendall's τ for 1,000 replications between the ordering based on the population test-score reliability and the ordering produced by the three item-score reliability methods and the corrected item-total correlation (CITC), for the bottom-up and the top-down procedure in the six different conditions.

	***N* = 200**			***N* = 1, 000**		
	**Small variance**	**Average variance**	**Large variance**	**Small variance**	**Average variance**	**Large variance**
	**of** **α**	**of** **α**	**of** **α**	**of** **α**	**of** **α**	**of** **α**
**BOTTOM-UP PROCEDURE**						
Method MS	0.44 (0.13)	0.67 (0.08)	0.80 (0.06)	0.69 (0.08)	0.83 (0.05)	0.89 (0.04)
Method λ_6_	0.55 (0.11)	0.75 (0.07)	0.83 (0.05)	0.80 (0.05)	0.91 (0.03)	0.94 (0.03)
Method CA	0.58 (0.10)	0.78 (0.06)	0.87 (0.05)	0.81 (0.05)	0.92 (0.03)	0.96 (0.02)
CITC	0.58 (0.10)	0.78 (0.06)	0.87 (0.04)	0.81 (0.05)	0.92 (0.03)	0.96 (0.02)
**TOP-DOWN PROCEDURE**						
Method MS	0.46 (0.14)	0.64 (0.10)	0.75 (0.08)	0.61 (0.11)	0.73 (0.09)	0.80 (0.08)
Method λ_6_	0.55 (0.10)	0.75 (0.07)	0.83 (0.06)	0.81 (0.05)	0.91 (0.03)	0.94 (0.03)
Method CA	0.59 (0.10)	0.78 (0.06)	0.87 (0.05)	0.81 (0.05)	0.92 (0.03)	0.96 (0.02)
CITC	0.59 (0.10)	0.78 (0.06)	0.87 (0.04)	0.81 (0.05)	0.92 (0.03)	0.96 (0.02)

*The Standard Deviation is in Parentheses*.

For the bottom-up item selection method (upper part) and the top-down item selection method (lower part), for each of the four item-assessment methods in the six different conditions, Table 5 shows Kendall's *W*-values. For both item selection methods, larger sample size showed an increase of *W*, indicating that the ordering was more alike across replications as sample size increased. This result was also found for increasing variance of discrimination parameters.

**Table 5:** Kendall's *W* for 1,000 replications between the ordering based on the population test-score reliability and the ordering produced by the three item-score reliability methods and the corrected item-total correlation (CITC), for the bottom-up and the top-down procedure in the six different conditions.

	***N* = 200**			***N* = 1, 000**		
	**Small variance**	**Average variance**	**Large variance**	**Small variance**	**Average variance**	**Large variance**
	**of** **α**	**of** **α**	**of** **α**	**of** **α**	**of** **α**	**of** **α**
**BOTTOM-UP PROCEDURE**						
Method MS	0.53	0.79	0.90	0.80	0.92	0.96
Method λ_6_	0.65	0.87	0.92	0.91	0.97	0.98
Method CA	0.69	0.89	0.95	0.91	0.97	0.99
CITC	0.69	0.89	0.95	0.91	0.97	0.99
**TOP-DOWN PROCEDURE**						
Method MS	0.37	0.65	0.80	0.61	0.77	0.85
Method λ_6_	0.53	0.82	0.89	0.88	0.96	0.98
Method CA	0.59	0.85	0.93	0.88	0.96	0.98
CITC	0.59	0.85	0.93	0.88	0.96	0.98

For both procedures, method CA and the corrected item-total correlation showed the highest *W*-values, suggesting the smallest variance of the orderings over data sets for these item-assessment methods. For the average and large variance of discrimination parameters, *W*-values were all >0.96, meaning that methods λ_6_, CA, and the corrected item-total correlation showed almost no variation over replications. For a large sample size, methods λ_6_, CA, and the corrected item-total correlation showed similar Kendall's *W*-values.

**Figure 1:**
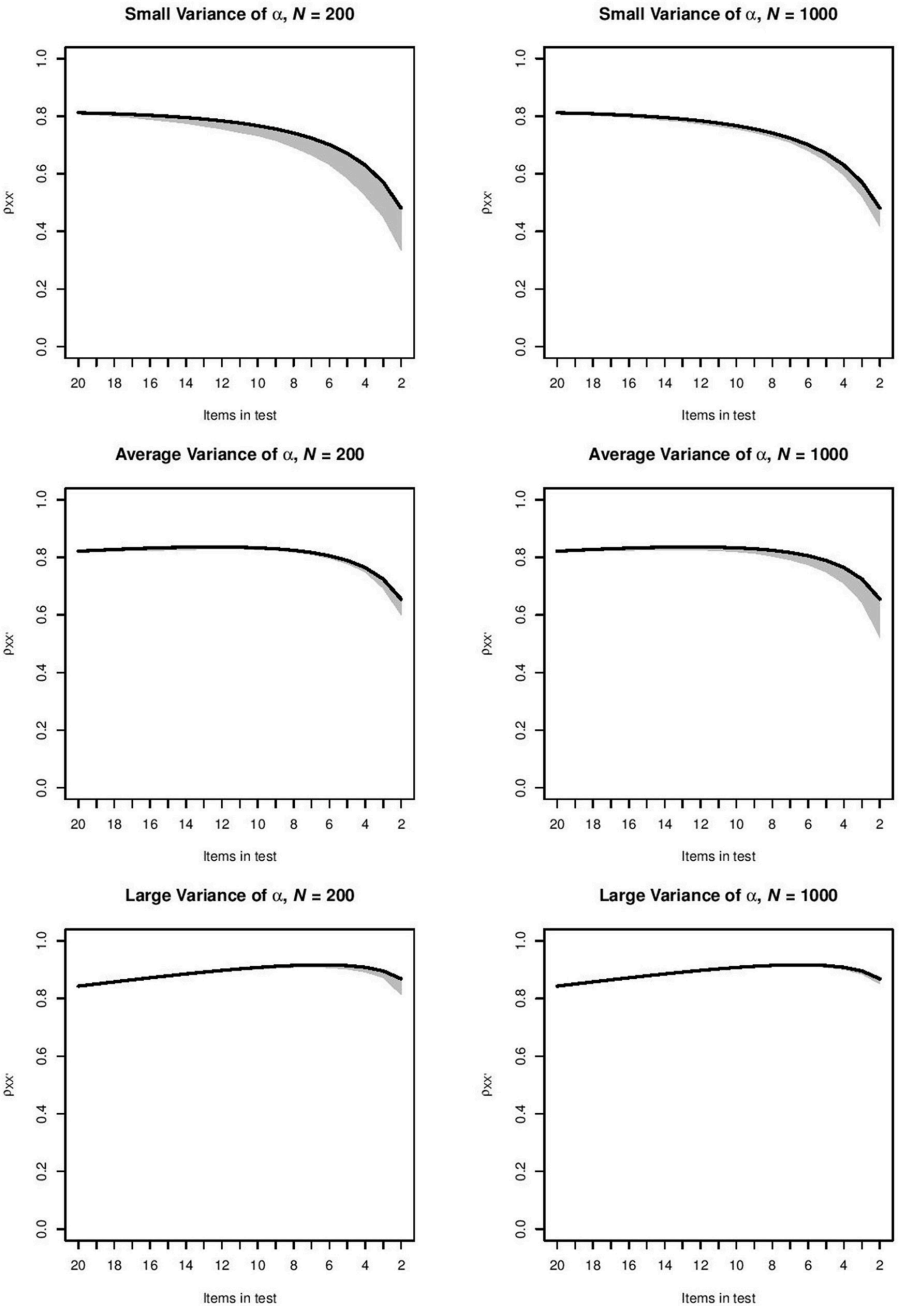
Range of $\rho_{XX^{\prime }}$-values between the 2.5 and 97.5 percentiles of 1, 000 values produced by method CA in the six conditions for the top-down procedure. The black line indicates the $\rho_{XX^{\prime }}$-value for the ideal ordering.

**Figure 2:**
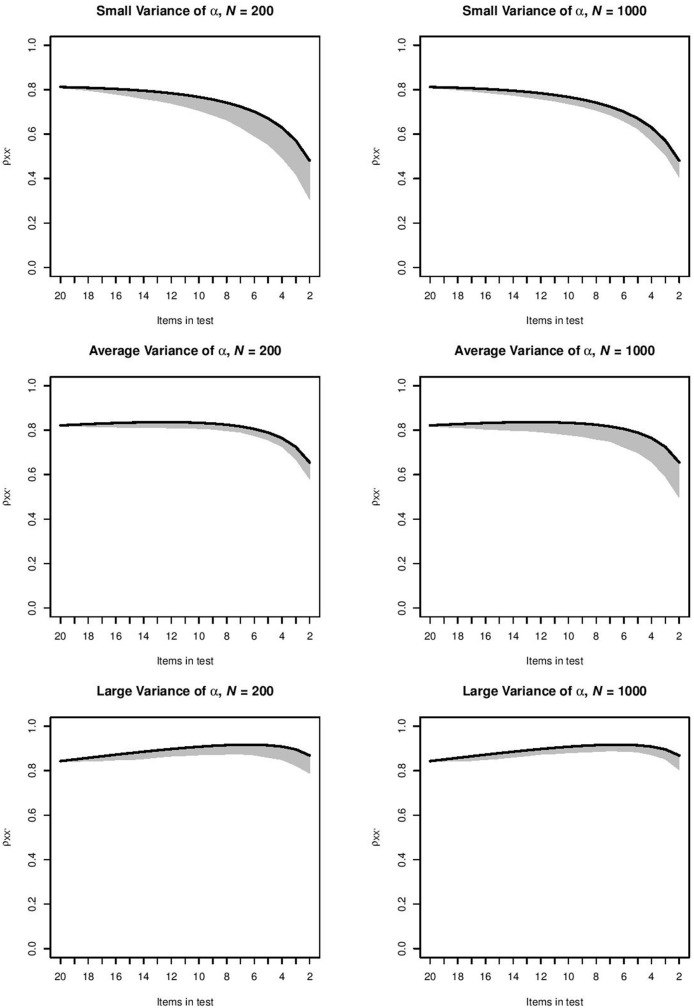
Range of $\rho_{XX^{\prime }}$- values between the 2.5 and 97.5 percentiles of 1, 000 values produced by method MS in the six conditions for the top-down procedure. The black line indicates the $\rho_{XX^{\prime }}$-value for the ideal ordering.

For each combination of item selection procedure and item-assessment method, the orderings produced by the item-assessment method were used to compute the $\rho_{XX^{\prime }}$- values in every step of this ordering. This meant that for the items selected at a particular step, we used the item parameters and the distribution of θ to compute $\rho_{XX^{\prime }}$, and we repeated the computation at each selection step in each of the 1, 000 samples. For the top-down item selection procedure and item-assessment method CA, Figure 1 shows for each step the range of $\rho_{XX^{\prime }}$-values between the 2.5 and 97.5 percentiles of 1, 000 values. This combination of item selection procedure and item-assessment method produced the intervals that were narrowest. Intervals became wider as the test grew shorter. For the top-down procedure and item-assessment method MS, Figure 2 shows the widest intervals. In both Figures 1, 2, intervals grew wider as the test grew shorter.

## 4. Discussion

This study investigated the usefulness of item-score reliability methods to select items with the aim to produce either a longer or a shorter test. In practice, a test constructor may aim at a particular minimally acceptable test-score reliability or a maximally acceptable number of items. The results showed that the benchmark corrected item-total correlation was the best item-assessment method in both the bottom-up and top-down item selection procedure. This means that the frequently employed and simpler corrected item-total correlation is, next to method CA, one of the best item-assessment methods when constructing tests.

Because method CA computes the item-score reliability using the corrected item-total correlation (Equation 3), it is not surprising that these two item-assessment methods showed nearly identical results. Given these identical results, using the corrected item-total correlation seems more obvious in practice than using method CA, because the corrected item-total correlation is readily available in most statistical programs and using method CA would merely introduce a more elaborate method. However, this does not mean that item-score reliability does not contribute to the test construction process. Method CA is an estimation method to approximate item-score reliability, while the corrected item-total correlation expresses the correlation between an item and the other items in the test. Method CA was developed to estimate item-score reliability, and to this means uses the corrected item-total correlation. The corrected item-total correlation is used to express the coherence between an item and the other items in a test. This means that these two measures were developed and are used with a different purpose in mind.

In our study, once an item was selected for addition to the test or removal from the test, the selection result was irreversible. An alternative stepwise procedure might facilitate adding items to the test with the possibility of removing them again later in the procedure or removing items from the test with the possibility of adding them again later in the procedure. An alternative stepwise item selection procedure in combination with the item-assessment methods may produce an ordering closer to the ideal order than the bottom-up or top-down procedures. Such procedures are the topic of future research. Also, the frequently used assessment method “coefficient alpha if item deleted” was not considered in this study. This assessment method would be easily applicable in the top-down item selection procedure, but for the bottom-up item selection procedure we would have to come up with something like “coefficient alpha if item added.” However, because the scope of this study was to investigate the construction of tests using assessment methods at the item level, and because coefficient alpha is on the test-level, we did not consider this assessment method. Also, we only studied one-dimensional data, because a test is assumed to measure one attribute. Deviations from one-dimensional data, which are unavoidable in practice because measurement in psychology is prone to systematic error, are the topic of future research.

Even though item-score reliability did not turn out to be a better item assessment method than the frequently employed corrected item-total correlation, it still has many useful applications. For example, when selecting a single item from a pool of items for constructing a single-item measure, item-score reliability can be used to ensure that the selected item has high item-score reliability. Single-item measures are often used in work and organizational psychology to asses job satisfaction (Zapf et al., 1999; Harter et al., 2002; Nagy, 2002; Saari and Judge, 2004; Gonzalez-Mulé et al., 2017; Robertson and Kee, 2017) or level of burnout (Dolan et al., 2014). Single-item measures have also been assessed in marketing research for measuring ad and brand attitude (Bergkvist and Rossiter, 2007) and in health research for measuring, for example, quality of life (Stewart et al., 1988; Yohannes et al., 2010) and psychosocial stress (Littman et al., 2006). Also, in person-fit analysis item-score reliability can be applied to identify items that contain too little reliable information to explain person fit (Meijer and Sijtsma, 1995). This leaves many useful applications for item-score reliability.

## Author Contributions

EZ and JT came up with the design of the study. EZ carried out the simulation study and drafted a first version. KS and LvdA provided extensive feedback, both on the study design and the manuscript. EZ revised the manuscript and JT, LvdA, and KS provided final feedback, on the basis of which EZ revised the manuscript again.

### Conflict of Interest Statement

The authors declare that the research was conducted in the absence of any commercial or financial relationships that could be construed as a potential conflict of interest.
